# Gene Regulations upon Hydrogel-Mediated Drug Delivery Systems in Skin Cancers—An Overview

**DOI:** 10.3390/gels8090560

**Published:** 2022-09-02

**Authors:** Ramya Mathiyalagan, Anjali Kariyarath Valappil, Deok Chun Yang, Se Chan Kang, Thavasyappan Thambi

**Affiliations:** 1 Graduate School of Biotechnology, College of Life Sciences, Kyung Hee University, Yongin-si 17104, Gyeonggi-do, Korea; 2 Department of Biopharmaceutical Biotechnology, College of Life Science, Kyung Hee University, Yongin-si 17104, Gyeonggi-do, Korea

**Keywords:** skin cancer, hydrogel, signaling mechanisms, pathways, gene regulation, MAPK pathway

## Abstract

The incidence of skin cancer has increased dramatically in recent years, particularly in Caucasian populations. Specifically, the metastatic melanoma is one of the most aggressive cancers and is responsible for more than 80% of skin cancer deaths around the globe. Though there are many treatment techniques, and drugs have been used to cure this belligerent skin cancer, the side effects and reduced bioavailability of drug in the targeted area makes it difficult to eradicate. In addition, cellular metabolic pathways are controlled by the skin cancer driver genes, and mutations in these genes promote tumor progression. Consequently, the MAPK (RAS–RAF–MEK–ERK pathway), WNT and PI3K signaling pathways are found to be important molecular regulators in melanoma development. Even though hydrogels have turned out to be a promising drug delivery system in skin cancer treatment, the regulations at the molecular level have not been reported. Thus, we aimed to decipher the molecular pathways of hydrogel drug delivery systems for skin cancer in this review. Special attention has been paid to the hydrogel systems that deliver drugs to regulate MAPK, PI3K–AKT–mTOR, JAK–STAT and cGAS-STING pathways. These signaling pathways can be molecular drivers of skin cancers and possible potential targets for the further research on treatment of skin cancers.

## 1. Introduction

**Figure 1:**
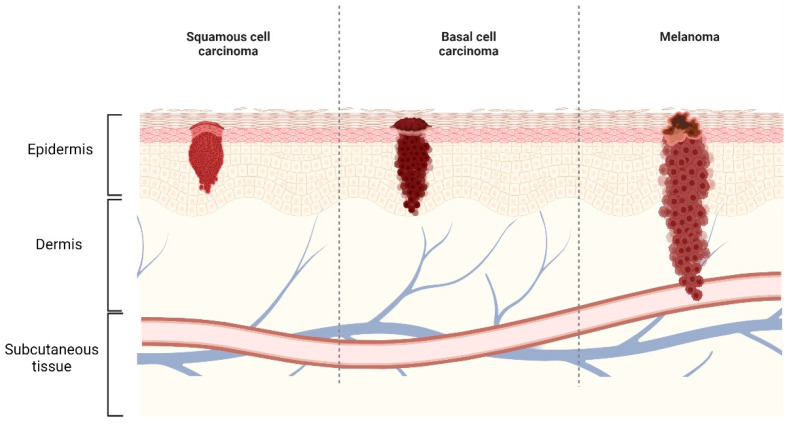
Different layers of the skin and types of skin cancer.

Skin is one of the largest and complex organs in the human body and it has some distinct functions such as acting as a protective fence in defense against injury due to UV radiation, chemicals and infections by microorganisms, enables the ability to feel, adjust body temperature and sensation [1]. Structurally, skin is a multi-lamellar structure, and the layers are epidermis, dermis, and subcutaneous tissue (Figure 1). The outermost layer, i.e., epidermis, is composed of the stratum corneum (SC) and the viable epidermis. The SC is metabolically inactive and possesses 10–24 layers of non-viable, elongated corneocytes (keratinized). These corneocytes are found to be embedded in the lipid bilayer matrix and this structural arrangement is known as the “Brick and Mortar” arrangement [2]. The extracellular lipid is composed of crystalline and liquid lipid phases. Hence, the skin acts as a principal physiological barrier inhibiting the uptake of polar compounds with high molecular weight (>500 Da) [3]. The dermis is thicker (3–5 mm) than the epidermis and comprises collagen fibrils and elastic connective tissues. Dermis consist of fibroblasts, mast cells, macrophages, lymphocytes, and melanocytes along with blood vessels, nerves, sweat and sebaceous glands [4]. Structurally, dermis does not show the same resistance to drug penetration as the SC, however, permeation of lipophilic drugs may be reduced in this layer. The subcutaneous tissue is a specialized fat cell layer, inter-connected by collagen and elastin fibers. Large quantities of fat are produced and stored in this layer [5].

To date, many reviews have been done on the applications against skin cancers [6,7]. However, the information on the regulations of the hydrogel drug delivery system of the drugs on the molecular mechanisms and pathways-related skin cancers is limited. This article provides a brief about skin cancer and its molecular drivers, and current treatment drugs along with an emphasis on studies conducted on hydrogel delivery system regulating gene expressions in skin cancers.

## 2. Skin Cancer

Skin cancer is the most common cancer among Caucasian populations and according to U.S. estimates, almost one in five Americans will develop some kind of skin cancer. DNA damage caused by ultraviolet (UV) radiation, followed by failure of DNA damage repair mechanisms, are the primary source of these neoplasms, and Fitzpatrick skin type and immunosuppression are also considered as potential risk factors for the development of skin cancer [8]. Skin cancers will develop in the outer most layers of the skin in the early stage and, if not treated, they may invade deeper into the skin with metastatic (secondary malignant growth far away from the primary origin) potential [7,9]. Based on their origin, skin cancers have been broadly classified into two types: non-melanoma skin cancer (NMSC) and melanoma.

### 2.1. NMSC

NMSCs are known to be the most common human cancers and UV radiation is the primary cause of them. UV-A (320–400 nm), UV-B (280–320 nm) and UV-C (100–280 nm) are the three regions of the UV spectrum. However, UV-B is considered as the most carcinogenic radiation and long term exposure to UV-B radiation will result in specific mutations in keratinocytes followed by NMSC [10]. Basel cell carcinoma (BSC) is the most common skin neoplasm, which develops in the basal layer of epidermis and is usually located on the face or the back of hands. Squamous cell carcinoma (SCC) is known to be the second most common skin cancer, develops in the squamous (spinosum) layer of epidermis and occurs mainly on neck and head areas (Figure 1). BCC shows slow growth and spreads locally with little or no metastasis, however, SCC may advance to invasive SCC and risk of metastasis is 2 to 6% [11,12]. Normally, earlier surgical removal of the metastatic NMSC and precancerous superficial lesions may inhibit its further progression into tumors.

Apoptosis is a crucial molecular mechanism in the development of malignancies in almost all type of cancers and an important cell survival pathway. The NFκB (nuclear factor kappa-light-chain-enhancer of activated B cells), Bcl-2, p53, TNF-related apoptosis-inducing ligand (TRAIL), ubiquitin ligases, overexpression of COX-2, mitogen-activated protein kinase (MAPK/ERK) pathways have significant role in skin cancers. The NF-κB regulates different cell survival pathways which control cell growth. Clinical studies have proven that disrupting NF-κB signaling in the epidermis causes terminal differentiation and promotes the appearance of SCC [13]. Bcl-2 and Bcl-x shows the overexpression in BCCs and SCC.

The p53 gene, known as the “guardian of the genome” is a member of the Bcl-2 protein family which found mutated in most of the human cancers. Mutations in the p53 gene induced by UV radiation has been regarded as a critical factor for developing skin cancer, as the susceptibility to apoptosis reduction would favor survival and tumor formation of mutated keratinocytes. Mutations of p53 are found in majority of NMSCs (more than 90% in SCC and 50% in BCC). Skin cancer cells cannot express Fas, but they can simultaneously express FasL, and thereby infiltrate antitumor T cells that express Fas will be killed. Reduction in the expression of the death receptor for TRAIL (a ligand that induces apoptosis from TNF family) was also observed in malignant cells in SCC [13,14,15]. The extracellular ligand-binding domain of epidermal growth factor receptor (EGFR) can bind to different ligands, i.e., epiregulin, EGF and TGF. Mutations in the EGFR tyrosine kinase domain activates the antiapoptotic signaling pathways in PI3K/AKT, ERK/MAPK and JAK–STAT [16]. Agustí et al. evaluated EGFR amplification deviations majorly in SCC [17].

Variations in the MAPK ERK signaling pathway have been detected in SCC. In non-transformed epidermis, Ras and Raf inhibit differentiation, stimulate cell division, and increase the expression of integrins [16]. In that K-RAS and H-RAS shows lower and higher level of mutations in SCC [13]. Despite the low rate of mutant RAS genes, an increase in levels of Ras with active GTP is observed in most tissues of spontaneous human SCC.

### 2.2. Melanoma

Melanoma is the deadliest skin cancer, originating from melanocytes, that predominantly affects younger and middle-aged people. Melanoma is not only developed on the skin, but also develops in the eyes, vagina, anus, sinus and oropharynx. However, occurrence in these areas consists of only 5% of total melanoma incidents. Cutaneous melanomas are classified as superficial spreading, lentigo malignant, nodular and acral lentiginous [18]. Because of their altering presentations, it is not easy to classify malignant melanomas. Though the incidence of melanoma is least, its often associated as an aggressive cancer condition for death, with increased tumor cell invasion and migration to other organs in the metastatic stage.

Various factors such as genetic and environmental factors such as prolonged sun exposure and sun burns by UV irradiation, lower melanin pigments, heredity, aging [10], more melanocytic nevi and immunosuppression in post-transplant patients can induce melanoma. The transformation of melanocytes (melanin producing cells present in the epidermal layer) into melanoma cells is a multistage process by occurrence of genomic alterations [19]. During the growth phase of the melanoma cells, it invades into the dermis/subcutaneous tissues followed by penetration into the capillaries and eventually enters the blood circulation to facilitate distant metastasis. The genetic mutations such as deletions, amplification, DNA methylation and translocations which drive melanoma were identified by genome-wide sequencing [20]. Therefore, the important genes that are known to be altered/mutated in melanoma and the molecular pathways involved in melanomagenesis are summarized in Table 1. Even though key genetic drivers are required for melanomagenesis, key microenvironmental factors play vital roles in modulating melanomagenesis and progression.

The MAPK pathway is most regularly triggered in cancer to facilitate rapid proliferation of tumor cells. Intracellular sequential activation of Ras, Raf, MEK, and ERK take place in with regard to extracellular binding of growth factors to receptor tyrosine kinases (RTKs) to regulate many oncogenic biological activities. The v-Raf murine sarcoma viral oncogene homologue B1 (BRAF) is one of the best-studied oncogenic mutations in melanoma. It encodes a serine/threonine protein kinase, a key regulator in the RAS–RAF–MEK–ERK MAPK pathway. Point mutation of BRAF by the substitution of valine to glutamic acid at codon 600 (V600E) occurs frequently (more than 50%) in melanoma than other types, which leads to a downstream MAPK pathway [47]. The wild-type BRAF melanomas have oncogenic mutations in upstream components of the MAPK pathway, such as NRAS (neuroblastoma RAS viral oncogene homolog), KIT (v-Kit Hardy–Zuckerman 4 feline sarcoma viral oncogene homolog), GNAQ (guanine nucleotide-binding protein, q polypeptide) and GNA11 (guanine nucleotide-binding protein, a11) [19].

Uncontrolled cell cycle is an important characteristic of melanoma development and p16^INK4A^ is a key down-regulator of the cell cycle, which is induced by the expression of oncogenic BRAF^V600E^. CDKN2A locus encodes p16^INK4A^ and is found mutated in 25% of melanoma types. The initiation of p16^INK4A^ by the MAPK pathway along with activation of microphthalmia-associated transcription factor (MITF) locus amplification is found in 20–30% of melanomas [48]. Triggering of the PI3K (phosphoinositide 3-kinase)–AKT–mTOR (mammalian target of rapamycin) pathway occurs constitutively in BRAF-initiated melanogenesis which inactivate PTEN (phosphatase and tensin homolog), a down-regulator of this pathway [26,27]. Though NRAS and PTEN mutations are mutually exclusive in melanomas, an oncogenic RAS also can trigger the PI3K–AKT–mTOR pathway. By contrast, even though mutations in the catalytic subunit of PI3K, or AKT1, AKT2, and AKT3, are rare in melanoma, immunopositivity of AKT3 is common in melanoma, and could activate PI3K–AKT–mTOR in PTEN wild type tumors. Amplification or mutations in CDK4 is another genetic alteration found in melanoma, which is the binding partner of p16INK4a. Hyperactivation of ERK or loss of p16INK4A can dysregulate the CDK4 pathway. Both CDKN2A and CDK4 have important roles in controlling cell cycle, as both mutate, which disturbs the G1/S-phase checkpoint [49,50].

### 2.3. Current Treatments and Drugs for Skin Cancers

Early diagnosis and immediate treatment are important in any type of skin cancer. Surgical and non-surgical treatments such as topical therapies are mostly carried out for skin cancer lesions [51]. Depending on the cancer progression stage, skin cancers can be treated by surgery [52], immunotherapy [53], cryosurgery [54], laser therapy [55], curettage, desiccation [56], dermabrasion [57], targeted therapy, photodynamic therapy [58], chemotherapy and radiotherapy [59]. Commonly used medications against melanoma include 5-fluorouracil (5-FU, Efudex), Imiquimod, Resiquimod, Ingenol mebutate, Diclofenac, cisplatin, etc. (Table 2). However, these conventional treatment methods and drugs can induce side effects such as hypopigmentation, scars, loss of hair, edema, gastrointestinal irritabilities such as chronic ulcer formation, blister formation and radiodermatitis.

SC acts as a major barrier to the penetration and delivery of adequate concentration of anti-skin cancer drugs to the targeted site when administrated topically. Many drugs that are currently used to treat cancer have a limited distribution in the tumor area and there are multiple factors, such as solid tumor development far away from the blood vessels, extracellular matrix composition, and elevated interstitial fluid, which cause the limited drug distribution [76]. Tumor microenvironment is a highly dynamic network of cells and molecules that create a favorable condition for the development of tumors. Immunosuppressive cells such as regulatory T lymphocytes and tumor-associated macrophages are general characteristic of the tumor microenvironment that are associated with extracellular matrix destruction, angiogenesis and metastasis [77,78]. The immune system of hosts has significant importance in response against cancer. However, most of the common cancer therapies that are used at present have immunosuppressive effects. For instance, myelosuppression is a common cause of chemotherapy and ionizing radiation [58,79]. Even though there are different immunotherapeutic techniques such as cytokines and monoclonal antibody-based therapies, antitumor vaccines and T cell therapies for treating SKs, disadvantages such as systemic side effects and cost of the therapies make immunotherapies still in their infancy [80].

## 3. Hydrogel: Promising Drug Delivery Systems to Treat Skin Cancers

To overcome the side effects associated with anti-neoplastic drugs in skin cancer treatment, hydrogel-based drug delivery systems could be promising drug candidates [81,82,83,84,85]. The hydrogels are three-dimensional, highly crosslinked polymers that can retain a substantial quantity of water in their swollen state due to the hydrophilic –OH, –CONH–, –CONH_2_, –COOH, and –SO_3_H groups [86,87,88,89]. Classification of hydrogels can be done based on the nature of material (synthetic, natural or hybrid) [90], mechanism of gel formation (chemically or physically crosslinked) [91], nature of side group (cationic, anionic or neutral) [92,93], biodegradability (degradable or non-degradable) [94,95], and the degree of swelling (low, high or superabsorbent) [96] and porosity (micro-, macro- or super porous) [97]. Hence, the compositions and synthesis conditions determine hydrogel properties and structures. For instance, highly porous structures permit more adherence and entrap the therapeutic agents in it, and the release of therapeutic agents can be controlled by regulating its porous structure [98,99,100]. These high swelling properties of biocarriers have a comparable degree of elasticity to natural tissues, and can undergo gel–solid phase transitions in response to various types of stimuli such as temperature, light, pressure, electric field, magnetic field, ionic strength and pH [101,102,103]. The natural polymers have reached a considerable significance in drug delivery applications, due to their characteristics such as biocompatibility, biodegradability, bifunctionality, biochemical stability, improved drug solubility, controlled drug release, cost effectiveness and nontoxicity [104,105,106,107]. These advantages, identical to the native extracellular matrix (ECM), and tunable physical and mechanical properties, aid in a vast variety of biomedical applications [108,109,110].

Unique characteristics of hydrogel such as tunable porous structure, high strength and stimulus response have made them widely used as carriers or vehicles for different biomolecules such as drugs, nucleic acids, antibodies, metal ions and enzymes, and are reported to be effective against different types of cancers such as lung cancer [111], leukemia [112], colon cancer [113], breast cancer [114], melanoma [115,116], hepatocellular carcinoma [116,117], etc. In addition, it is applied in the delivery of antibodies and other immune modulatory molecules at tumor sites in immunotherapy [118], drug carriers for the eye [119] and drug-delivering contact lenses for glaucoma therapy, in tissue engineering and mesenchymal stem/stromal cells [120]. Hence, it has been used to treat osteoporosis [121], osteoarthritis [122] and wound healing [123]. The topical and transdermal drug delivery using hydrogel is a convenient way to deliver the drugs systemically, in which the drug penetrates through the SC initially and to the epidermis and dermis for systemic absorption via dermal microcirculation [124]. Hence, it has many advantages such as increased patient compliance by reduced dosing, prevents pre-systemic metabolism for enhanced bioavailability [125], and soft swollen hydrogels [126]. In contrast to topical treatment, injectable hydrogels are generally introduced into the body via syringe or catheter. Since the blood circulation will quickly remove the normal chemotherapeutics injected inside the body, the effect of drugs will not be enough to kill the cancer cells. Therefore, engineering injectable hydrogels by physical or chemical cross-linking for sustained and controlled drug release at in situ (near cancer) upon minimal injection enables higher drug concentration at the targeted site while diminishing the systemic drug concentration and the associated site effects. Moreover, the drugs can be delivered into tissues that are difficult to access through surgery [127,128]. Hydrogel particles are transported to the intercellular matrix through plasma membrane (PM) during drug or gene delivery and PM act as a major obstacle for the efficient delivery. The small or macro molecules enter the cells by endocytosis and target specific organelles for efficient drug delivery. Hydrogels are mainly internalized through endocytosis with clathrin- and dynamin-dependent pathways [129]. The physiochemical properties such as shape, size, charge and chemistry of the surface of colloidal particles determine the cell translocation and intracellular distribution [130,131,132]. Particles with nanometer to micron sizes are generally subjected to intercellular uptake. However, particles with a diameter of around 100 nm are known to show a higher degree of uptake. For example, particles with a 100–200 nm size range avoided premature clearance by the reticuloendothelial system in cancer cells [133,134]. The smaller particles are internalized into B16F10 murine melanoma cells through clathrin-dependent pathway and microtubule dependent intracellular trafficking whereas larger particles are internalized through caveolae mechanism [135].

## 4. A Brief Background on the Use of Hydrogel-Based Drug Delivery System to Regulate Skin Cancer Related Genes

**Figure 2:**
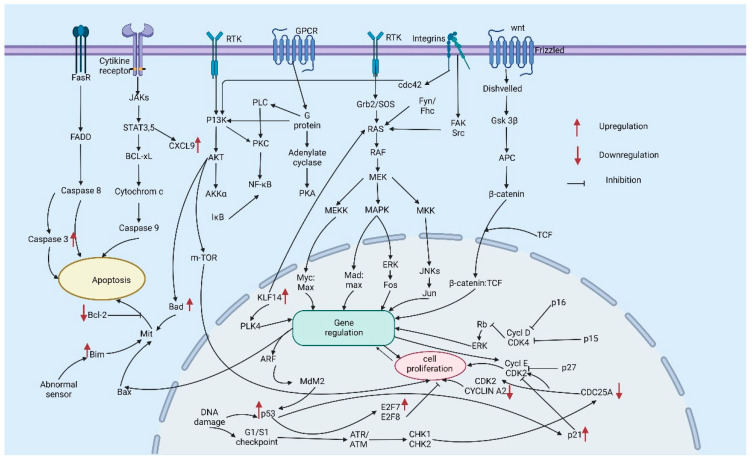
Gene regulations on hydrogel mediated drug delivery treatment for skin cancers, based on the recent reports. The upregulation of Caspase 3, Bim, BAD, CXCL9, KLF14, p53, p21 and downregulation of Bcl-2, CDK2, CDC25A were mainly reported after the hydrogel-based drug delivery for skin cancers as apoptosis and cell proliferation regulators.

Over the past decade, a plethora of chemotherapeutic agents has been developed or studied to challenge skin cancers and most of these agents remained unsuccessful, especially to eradicate melanoma. Because melanoma is considered to be intrinsically resistant to chemotherapeutics, and different mechanisms such as overexpression of drug efflux proteins, alteration of enzyme activation, deregulation of apoptosis, Ras mutation, epithelial to mesenchymal transition, and deregulation of microRNAs expression are responsible for this drug resistance [136]. There will be a significant change in the recovery rate if this drug resistance is conquered. Availability of trace amounts of drugs in the tumor microenvironment is one of the factors that contribute to sensitivity of drugs. Since tumor microenvironment is very complex and dynamic, it is very difficult to deliver chemotherapeutics in sufficient concentration. Although previous studies have reported the need of an efficient advanced drug delivery system to overcome this problem, microsphere or nanomaterial-based cancer eradication developed slowly. However, initial burst release, increased drug accumulation in the tumor and rapid elimination by the reticuloendothelial system have reduced the efficacy of nanoformulations to affect the cancer cells [137,138]. Hydrogels are potential carriers for localized delivery of anti-neoplastic agents. Blood supply and morphology of blood vessel network do not affect the hydrogel-based delivery system. Therefore, hydrogels can be utilized to carry drugs that are capable of killing cancer cells as well as to regulate the genes related to skin cancer and deliver in the targeted local tumor microenvironment. Because of molecular complexity of SK, combinatorial drug therapy is gaining more importance. In opposition to single-drug therapy, multiple-agent therapy can maximize the therapeutic effects, modulate cancer related signaling pathways, and overcome drug resistance. In addition, combinatorial drug therapies may be able to trigger senescence of cancer cells and permit clearance by T cells. Hydrogels can carry and deliver more than one therapeutic agent simultaneously to the targeted site [7,139,140,141]. To decipher the gene regulations mediated by hydrogel, Zhao, Y., et al. developed an injectable hydrogel loaded with Cripto-1 receptor antibodies (2B11) for embryonic microenvironments on tumor reversion treatment using B16 tumor-bearing mice model [142] in which cancer cell morphology reversed normal melanin cells. Additional RNA-sequencing experiments in comparison with the whole gene expression show that 2B11 hydrogels could significantly stimulate apoptosis. Oncogenes (Kit, Itga4, Hapln1) were downregulated and tumor suppressors genes (Irf8, Trail, Casp1, Aim2, Irf1) were upregulated compared with the control group. The interleukin-15 (IL-15)- and cisplatin (CDDP)-loaded poly (ethylene glycol)-poly(γ-ethyl-L-glutamate) diblock copolymers (mPEG-b-PELG) thermosensitive hydrogels injected into mice bearing B16F0-RFP melanoma cells exhibited synergistic immune regulations such as Cyclin A2, CDK2 and Cdc25A expression was significantly reduced [143]. Another co-delivery of doxorubicin and curcumin peptide hydrogel increased the inhibitory effect of cell growth and improved apoptosis by differential apoptotic/anti-apoptotic gene expression profiles in head and neck squamous cell carcinoma by Karavasili, C., et al. [144]. The various studies that have been carried out on hydrogel delivery systems regulating the molecular mechanisms of skin cancers are included in Table 3 and Figure 2.

## 5. Conclusions and Perspectives

Skin cancers are often non-cared but persist as the most common malignancy of humans and affect millions of people every year. Even though vast research on skin cancer has enriched our understanding of the disease, 100% eradication is yet to be achieved. The expanding knowledge of molecular mechanisms and pathways involved in NMSCs, and melanoma pathogenesis could lead to identify the potential molecular players. In addition, the hydrogel-based delivery systems with drugs were mainly regulated the MAPK, PI3K–AKT–mTOR, JAK–STAT and cGAS-STING pathways. Though very minimal studies have been conducted, these pathways could be a potential target for further researchers.

In the future, the skin cancers can be treated using various therapies such as chemotherapy, immunotherapy, gene therapy as well as combinatorial therapy. In particular, the advancement in the hydrogel-based drug delivery systems can minimize the side effects of the chemotherapeutic drugs. The sequencing and genome engineering technologies could be able to identify the potential markers in the skin cancer and could help to proceed gene based targeted delivery and therapy. As the p53 gene mutations have been a critical factor for developing skin cancer, the gene therapy could induce the expression of the p53 genes which could restore the apoptosis pathway and destroy the cancer cells. In addition, as the mutation of BRAF is one of the important oncogenic mutations that act as a key regulator in the RAS–RAF–MEK–ERK MAPK pathway in melanoma, development of the targeted therapy to inhibit these point mutations also could treat skin cancers. Moreover, receptor mediated targeted therapy and microbiome replacement therapy also a potential candidate to improve the treatment options.

## Figures and Tables

**Table 1:** Genes and pathways involved in melanoma.

Gene	Pathway	Regulation	References
BRAF	RAS–RAF–MEK–ERK mitogen-activated protein kinase (MAPK) pathway	Somatic missense mutation-valine-to-glutamic acid substitution at position 599 (V599E)	[21]
NRAS	RAS–RAF–MEK–ERK MAPK pathway	Substitution mutations	[22]
KIT	mitogen-activated protein (MAP) kinase and phosphatidylinositol 3 (PI3) kinase pathways, PI3K–AKT–mTOR, JAK–STAT	Somatic mutations- on exon 11 (L576P or exon 13 (K642E)	[23,24]
GNAQ	MAPK pathways	Somatic mutations—glutamine at position 209 (Q209) is mutated to either leucine or proline	[25]
GNA11	MAPK pathway	Somatic mutations—glutamine at position 209 (Q209) is mutated to either leucine or proline	[26]
CDKN2A (p14^ARF^)	MDM2–p53	Deletion	[27,28]
CDKN2A (p16^INK4A^)	p16^INK4A^–cyclin D/CDK4–RB checkpoint	‘G’ TO ‘A’ transition at the first nucleotide of the splice donor site of intron 2	[28,29]
PTEN	oncogenic phosphatidylinositol-3-kinase (PI3K) signaling pathway	Deletion or mutation leads to constitutive activation of this pathway	[30]
LKB1	LKB1–AMPK	‘C’ to ‘T’ transition, resulting in the substitution of the normal glutamine codon (CAG) with a premature termination codon (TAG)	[31,32]
MITF	MITF–PGC1a Transcription, lineage, cell cycle	Amplification/germline missense substitution	[33,34]
NF1/neurofibromin	PI3K and MAPK pathways	Mutation	[35]
MYC		Amplification	[36]
Cyclin D1	RAS/MAPK pathways	Amplification	[37,38]
CDK4	Cell cycle, G1/S cyclin-dependent kinase	Amplification or Point mutation	[39]
HDM2	P53	Amplification	[40]
PIK3CA	PI3K–AKT–mTOR	Missense mutations	[41]
AKT1,AKT2, AKT3	PI3K–AKT–mTOR	Oncogenic mutation	[42]
ERBB4	Receptor tyrosine kinases	Amplification	[43]
fibroblast growth factor receptor 3 (FGFR3)	Ras/MAPK	Amplification, gain-of-function mutations	[44,45]
MET	PI3K, MAPK	Amplification/single-nucleotide variations	[46]

**Table 2:** Current drugs used for skin cancer.

Drug	Origin	Molecular Weight (g/mol)	Structure	Route of Administration	Effect	References
Alitretinoin	Synthetic/Natural	300.4	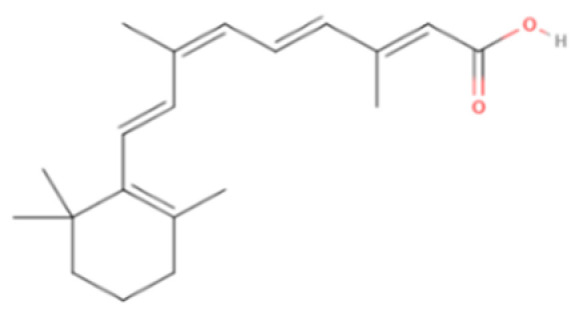	Topical	Inhibit cyclooxygenase (COX)-2 expression, suppress cell growth	[5,60]
Diclofenac sodium	Synthetic	318.1	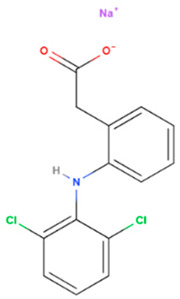	Topical	Cycloxygenase-2 enzyme overexpression & increase apoptosis	[61,62]
Fluorouracil	Synthetic	130.1	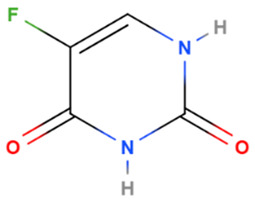	Topical	Enzyme responsible for synthesis of thymidine, one of the pyrimidine nucleosides of DNA has been inhibited	[5]
Imiquimod	Synthetic	240.3	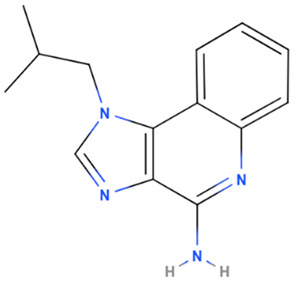	Topical	Through toll-like receptor 7 (TLR7) an immune response which upregulates cytokines and consequently leads to apoptosis has been triggered	[5,63]
Ingenol mebutate	Synthetic/Natural	430.5	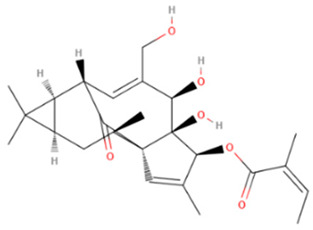	Topical	Upregulate rapid lesion necro sis and neutrophil-mediated cellular cytotoxicity, Modulation of protein kinase C isoforms	[64,65]
Cisplatin	Synthetic	301.1	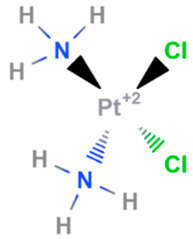	Intravenous	DNA cross linking agents	[66]
Dacarbazine	Synthetic	182.18	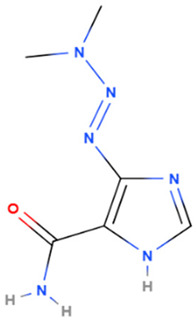	Intravenous	Alkylating agents that damage DNA by introducing alkyl groups to guanine bases, eventually causing cell death via apoptosis and other cell death mechanisms	[7,67]
Temozolomide	Synthetic	194.151	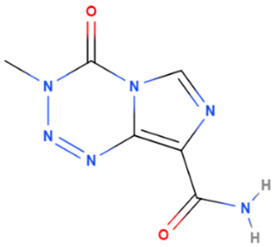	intravenous infusion	Alkylating agents that damage DNA by introducing alkyl groups to guanine bases, eventually causing cell death via apoptosis and other cell death mechanisms	[67]
Carmustine	Synthetic	214.05	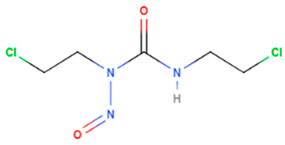	intravenous infusion	DNA binding and DNA alkylation by a nitrogenous base within a duplex	[68]
Lomustine	Synthetic	233.69	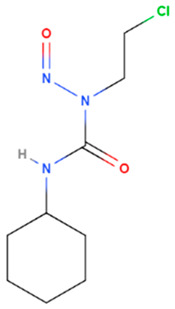	oral	Alkylation of the O^6^ position of guanine-containing bases in DNA and the enzyme O^6^ -alkylguanine transferase mediates	[69]
Vincristine	Natural	824.958	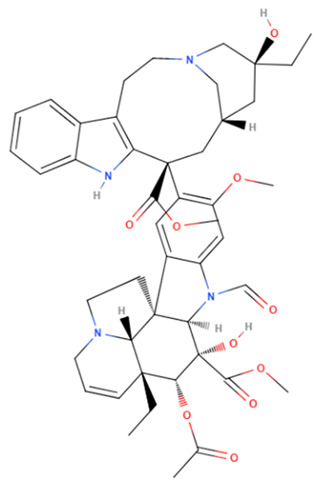	intravenous infusion	Inhibition of microtubule polymerization; microtubule destabilization	[70]
Vinblastine	Natural	810.974	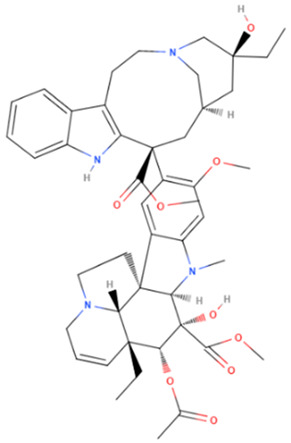	intravenous infusion	Inhibition of microtubule polymerization; microtubule destabilization	[70]
Carboplatin	Synthetic	371.249	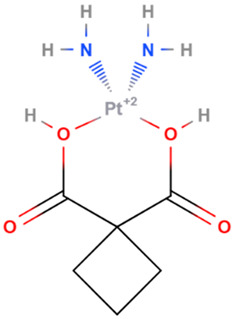	intravenous infusion	DNA alkylation: formation of DNA crosslinks and adducts; inhibition of DNA synthesis	[7,70]
Taxol/Paclitaxel	Natural	853.906	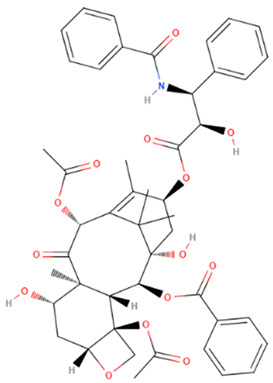	intravenous infusion	Interacts with microtubules causing polymerization and stabilization of the tubulin polymer which prevents successful completion of cell division	[71,72]
Docetaxel	Natural	807.879	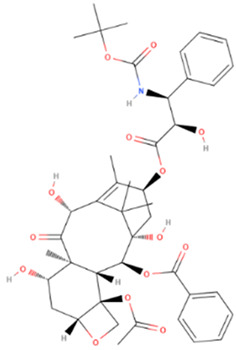	intravenous infusion	Binds to β-tubulins in the cell and causes their polymerization, induces caspase-2 dependent apoptosis	[73]
Resiquimod	Synthetic	314.4	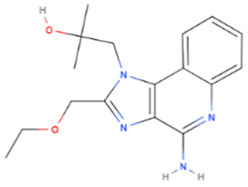	Topical	Stimulates immune responses through toll-like receptors (TLR) 7 and 8 dependent pathway activation	[74]
Mechlorethamine	Synthetic	156.05	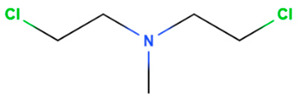	Topical	Cancer cell growth postulated by DNA cross-linking and depurination, or abnormal base paring.	[75]

**Table 3:** Recent studies on the gene regulations upon hydrogel-based drug delivery system for skin cancers.

Hydrogel	Gene	Pathway	Effect	In Vivo/In Vitro	Cancer	Reference
Hyaluronic acid scaffold loaded with Nodal Signaling Crypto 1 receptor antibodies (2B11)	Kit, Itga4, Hapln1, Irf8, Trail, Casp1, Aim2, and Irf1	Apoptosis pathways, MAPK pathways and PI3K–AKT–mTOR, JAK–STAT	Upregulation of tumor suppressor genes and the downregulation of oncogenes	In vivo and in vitro	Melanoma	[142]
mPEG-b-PELG hydrogel encapsulating f interleukin-15 (IL-15) and cisplatin	Cyclin A2, CDK2, and Cdc25A	Cell cycle	Significant decrease in the expressions of genes and cell cycle arrest	Ex vivo	Melanoma	[145]
Poly Lactic-co-Glycolic Acid (PLGA)-polyethylene glycol-PLGA hydrogel encapsulating, nano-hydroxyapatite and granulocyte-macrophage colony-stimulating factor	E2Fs family genes and PLK1, KLF14, KLF11	Cell cycle	Cell cycle arrested in the G2/M phase, apoptosis	In vitro	Melanoma	[143]
PELG-PEG-PELG loaded with DOX, IL-2, and IFN-g	Bcl-2 Caspase 3	Janus kinase, JAK/STAT and mitochondrial signal pathways	Induces apoptosis	In vitro	Melanoma	[146]
Olesterol-bearing cycloamylose with spermine group nanogel carrying VEGF-specific short interfering RNA	Vascular endothelial growth factor (VEGF)	Angiogenesis		In vitro		[147]
peptide hydrogel (ac-(RADA)4-CONH2) loaded with curcumin and doxorubicin	p53, p21, BAX, BAD, Cdk2, Bcl-2, c-myc and CyclD1	Apoptosis pathways,	High rate of apoptosis	In vitro	Head and neck squamous cell carcinoma	[144]
Alginate hydrogel bearing loaded with anti-PD-1 monoclonal antibody and celecoxib	IL-1b, IL-6, CXCL9 and CXCL10	Programmed death 1 (PD-1) signaling pathway	Increases the expression of two anti-angiogenic chemokines and suppresses the intra tumoral production of interleukin (IL)-1, IL-6, and cycloxygenase-2 (COX2)	In vitro	Melanoma	[148]
RADA24-melittin fusion peptide hydrogel loaded with cell-derived secretions from cells exposed to HOCl	IFN-α, IFN-β, IL-6	cGAS-STING pathway and PD-1 signaling pathway	Increased tumor cell death, cytotoxic T lymphocyte infiltration, and tumor-associated macrophage reprogramming towards an M1 phenotype	In vitro	Melanoma	[149]
N-succinyl chitosan and oxidized dextran hydrogel loaded with doxorubicin	CD206, Arginase-1, TNF-α and Inos	p65 NF-kappaB and P53 pathway	Induced macrophages to produce anti-tumorigenic cytokines such as TNF-α, iNOS, IL-6 and IL-1β	In vivo and in vitro	Melanoma	[150]
Polyvinyl alcohol/gelatin	mechanotransduction related genes and transposase-accessible chromatin	MAPK pathway and MKL1/SRF pathway	Poor cell adhesion and increased chromatin accessibility	in vitro	melanoma	[151]

## Data Availability

No new data were created or analyzed in this study. Data sharing is not applicable to this article.
